# Dr. Edward M. Moore

**Published:** 1918-06

**Authors:** 


**Dr. Edward M. Moore** of Rochester died April 5, age 67. He was graduated from the University of Buffalo in 1874.

